# A novel cell-based screen identifies chemical entities that reverse the immune-escape phenotype of metastatic tumours

**DOI:** 10.3389/fphar.2023.1119607

**Published:** 2023-05-15

**Authors:** Lilian L. Nohara, Samantha L. S. Ellis, Carola Dreier, Sarah Dada, Iryna Saranchova, Kyong Bok Choi, Lonna Munro, Cheryl G. Pfeifer, Eliana Al Haddad, Krysta M. Coyle, Jessica R. Morrice, Daniel Joo Sung Shim, Paul Ahn, Nicole De Voogd, David E. Williams, Ping Cheng, Emmanuel Garrovillas, Raymond J. Andersen, Wilfred A. Jefferies

**Affiliations:** ^1^ Michael Smith Laboratories, University of British Columbia, Vancouver, BC, Canada; ^2^ Centre for Blood Research, University of British Columbia, Vancouver, BC, Canada; ^3^ The Djavad Mowafaghian Centre for Brain Health, University of British Columbia, Vancouver, BC, Canada; ^4^ Department of Microbiology and Immunology, University of British Columbia, Vancouver, BC, Canada; ^5^ Vancouver Prostate Centre, Vancouver Coastal Health Research Institute, Vancouver, BC, Canada; ^6^ Departments of Medical Genetics, Zoology, and Urologic Sciences, University of British Columbia, Vancouver, BC, Canada; ^7^ Department of Molecular Biology and Biochemistry, Simon Fraser University, Burnaby, BC, Canada; ^8^ Department of Ophthalmology and Visual Sciences, University of British Columbia, Vancouver, BC, Canada; ^9^ Netherlands Centre for Biodiversity Naturalis, Leiden, Netherlands; ^10^ Departments of Chemistry and Earth Ocean and Atmospheric Sciences, University of British Columbia, Vancouver, BC, Canada

**Keywords:** antigen processing machinery, curcuphenol, major histocompatibility complex class I, metastatic tumours, natural products, high throughput cell-based assay, drug discovery

## Abstract

Genetic and epigenetic events have been implicated in the downregulation of the cellular antigen processing and presentation machinery (APM), which in turn, has been associated with cancer evasion of the immune system. When these essential components are lacking, cancers develop the ability to subvert host immune surveillance allowing cancer cells to become invisible to the immune system and, in turn, promote cancer metastasis. Here we describe and validate the first high-throughput cell-based screening assay to identify chemical extracts and unique chemical entities that reverse the downregulation of APM components in cell lines derived from metastatic tumours. Through the screening of a library of 480 marine invertebrate extracts followed by bioassay-guided fractionation, curcuphenol, a common sesquiterpene phenol derived from turmeric, was identified as the active compound of one of the extracts. We demonstrate that curcuphenol induces the expression of the APM components, TAP-1 and MHC-I molecules, in cell lines derived from both metastatic prostate and lung carcinomas. Turmeric and curcumins that contain curcuphenol have long been utilized not only as a spice in the preparation of food, but also in traditional medicines for treating cancers. The remarkable discovery that a common component of spices can increase the expression of APM components in metastatic tumour cells and, therefore reverse immune-escape mechanisms, provides a rationale for the development of foods and advanced nutraceuticals as therapeutic candidates for harnessing the power of the immune system to recognize and destroy metastatic cancers.

## Introduction

Despite recent advances, cancer remains a leading cause of death worldwide (Cancer Statistics, 2021). The majority of all cancers arise spontaneously and are initiated by genetic damage or cell deregulation, which is caused by changes in gene sequence and/or gene expression that lead to epigenetic alterations and structural variations (Ponder, 1992; Dada et al., 2023; Esteller, 2008; Lakshminarasimhan and Liang, 2016). Due to the increased rate of evolution seen in cancer cells, there is a greater chance for them to transition from a primary form into a more lethal metastatic form, which possess the ability to travel to distant sites and form secondary tumours (Mehlen and Puisieux, 2006). The metastatic form is of great concern because it is responsible for 90% of cancer deaths (Mehlen and Puisieux, 2006; Nguyen and Massagué, 2007b). Alterations associated with a cancer transitioning into a metastatic form are referred to as the “metastatic gene signature” (Jones and Baylin, 2002; Nguyen and Massagué, 2007b). Currently, there are several signatures associated with metastatic progression however one signature, loss of immunogenicity, habitually appears across several cancers (Jefferies et al., 1993; Gabathuler et al., 1994; Marusina et al., 1997; Gabathuler et al., 1998; Alimonti et al., 2000; Shankaran et al., 2001; Zhang et al., 2002; Vitalis et al., 2005; Lou et al., 2007; Zhang et al., 2007; Lou et al., 2008; Setiadi et al., 2008; Newman and Cragg, 2016a; Saranchova et al., 2016; Saranchova et al., 2018; Dada et al., 2023).

The endogenous antigen processing and presentation machinery (APM) allows T cells of the adaptive immune system to differentiate between normal cells, virus infected cells or cancerous cells (Jefferies et al., 1993; Zhang et al., 1999; Setiadi et al., 2008; Newman and Cragg, 2016a; Saranchova et al., 2016; Dhatchinamoorthy et al., 2021). The immune system is critical for maintenance of tissue homeostasis by acting as the primary defence against invading pathogens and damaged or cancerous cells (Jefferies et al., 1993; Zhang et al., 1999; Setiadi et al., 2007; Setiadi et al., 2008; Saranchova et al., 2016). It is frequently documented that under the selection of the host immune system, cancer cells establish mechanisms for evasion of the cell-mediated wing of the adaptive immune system to avoid destruction (Alimonti et al., 2000; Shankaran et al., 2001; Newman and Cragg, 2016a). This phenotype may be caused by one of several mechanisms, however, a recurrent defect seen in several cancers, especially metastatic, is the downregulation of the endogenous APM (Jefferies et al., 1993; Setiadi et al., 2007; Setiadi et al., 2008; Newman and Cragg, 2016a; Saranchova et al., 2018). MHC-I and TAP-1 downregulation in tumours leading to immune-evasion has been reported in many studies (Tomlinson and Bodmer, 1999; Alimonti et al., 2000; Setiadi et al., 2008; Cragg et al., 2012; Tur et al., 2021); however, the molecular mechanisms underpinning this phenomena remain the focus of current investigation (Dhatchinamoorthy et al., 2021). Overall, a significant body of work has shown that MHC-I antigen presentation commonly becomes defective in cancers in the transition from primary tumours to their metastatic forms and allows these cells to become invisible to tumour specific cytotoxic T lymphocytes and thereby evade immune surveillance (Gabathuler et al., 1994; Marusina et al., 1997; Gabathuler et al., 1998; Alimonti et al., 2000; Zhang et al., 2002; Vitalis et al., 2005; Lou et al., 2007; Zhang et al., 2007; Lou et al., 2008; Setiadi et al., 2008).

Nature is an important source for immunomodulators and anticancer compounds. The natural products paclitaxel, vincristine, doxorubicin, and bleomycin are among the most important anticancer drugs in clinical use (Cragg et al., 2012) and it has been estimated that between the 1940s and 2014, roughly 50% of all new FDA approved anticancer drugs were either natural products or derived from natural products (Newman and Cragg, 2016a). Marine organisms represent a highly biodiverse, but relatively unexplored, resource for the discovery of new natural product anticancer drug leads (Newman and Cragg, 2016b). The realization of the potential of this resource is illustrated by the clinically approved anticancer drugs Ara-C, Adcetris, Yondelis and Halavan, which are all based on natural products isolated from marine invertebrates. The marine invertebrate extract collection screened in this study has been a rich source of novel natural product chemical biology tools and drug leads (Loganzo et al., 2003; Karjala et al., 2005; Brastianos et al., 2006; Carlile et al., 2012; Stenton et al., 2013; Andersen, 2017) and, therefore, it was selected as an excellent resource for discovery of new compounds that may overcome immuno-evasion.

Here we describe the first cell-based screening assay that has been undertaken to identify chemical entities that reverse the phenotype of APM-deficiency in metastatic cancer cells. We validate this cell-based screening assay by discovering extracts and compounds from a library of marine invertebrate extracts that reverse the immune-escape phenotype in cell lines derived from metastatic tumours. We subsequently focus on the surprising finding that curcuphenol, a component of the culinary spices turmeric and curcumin used in traditional medicines and dietary supplements, induces the expression of APM components, TAP-1 and MHC-I, in both metastatic prostate and lung carcinoma cells.

## Results

### Selection of LMD pTAP-1 clone

**FIGURE 1:**
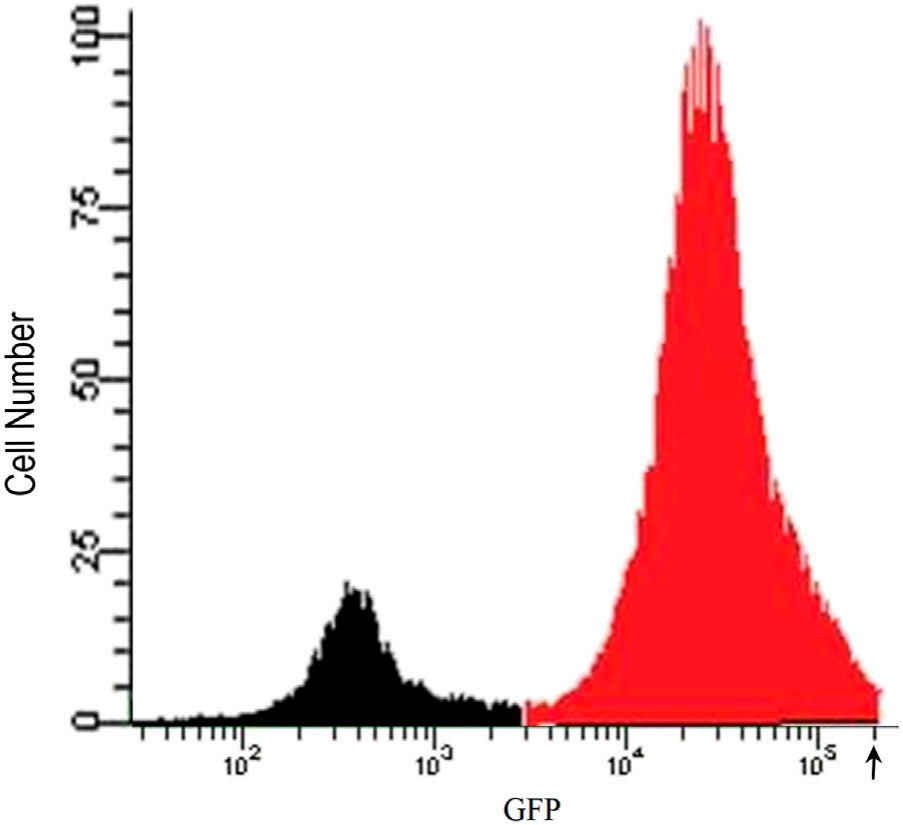
Flow cytometry analysis of IFN-γ stimulated unsorted LMD pTAP-1 metastatic prostate cancer cells expressing GFP under the TAP-1 promoter. LMD pTAP-1 cells were stimulated with 100 ng/mL of IFN-γ and analyzed by flow cytometry. Histogram shows GFP negative (black) and GFP positive (red) LMD pTAP-1 cells. Cells expressing high GFP levels (cells with fluorescence intensity above where the arrow is indicating) were sorted individually into 96-well plates to obtain clonal populations for further analysis.

**FIGURE 2:**
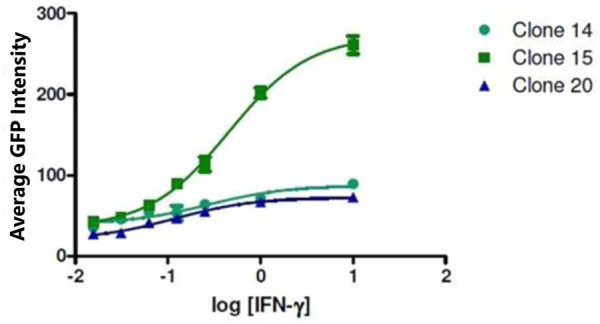
Selection of LMD pTAP-1 clone. The intensity of GFP fluorescence for clones 14, 15 and 20 is shown after stimulation of the LMD pTAP-1 cells with a range of IFN-γ concentrations. Clone 15 showed the highest TAP-1 promoter activity measured as GPF intensity after IFN-γ stimulation and was chosen for further experiments.

To develop a cell-based screening assay to identify chemical extracts and compounds that possess the ability to reverse the downregulation of APM components in cells from metastatic tumours, we used the LMD pTAP-1 metastatic prostate cell line. LMD pTAP-1 cells are a TAP-1 and MHC-I-deficient cell line that expresses enhanced green fluorescent protein (EGFP) under the TAP-1 promoter (Setiadi et al., 2005). In these cells, induction of TAP-1 promoter activity can be detected by measuring GFP fluorescence intensity. In order to obtain a clonal population of LMD pTAP-1 cells with low basal GFP expression and high GFP expression upon TAP-1 promoter induction, first, unsorted LMD pTAP-1 cells were stimulated with interferon-γ (IFN-γ), a known inducer of TAP-1 expression, and the cells with the top 2% highest GFP expression after stimulation (Figure 1) were individually sorted into 96-well plates to attain single cell clones. Upon clonal expansion and analysis of GFP fluorescence intensity, the LMD pTAP-1 clones number 14, 15 and 20 which showed low basal GFP expression, were selected. Then, these clones were stimulated with varying concentrations of IFN-γ to generate a dose-response curve (Figure 2). Clone 15 demonstrated the largest response to IFN-γ stimulation and was selected for further experiments. The selection of low GFP-expressing cells maximized the potential window between the positive and negative controls which is ideal for subsequent development of a high-throughput screening assay where a maximum difference between unstimulated and stimulated cells is vital.

### Development of high-throughput cell-based screening assay

**FIGURE 3:**
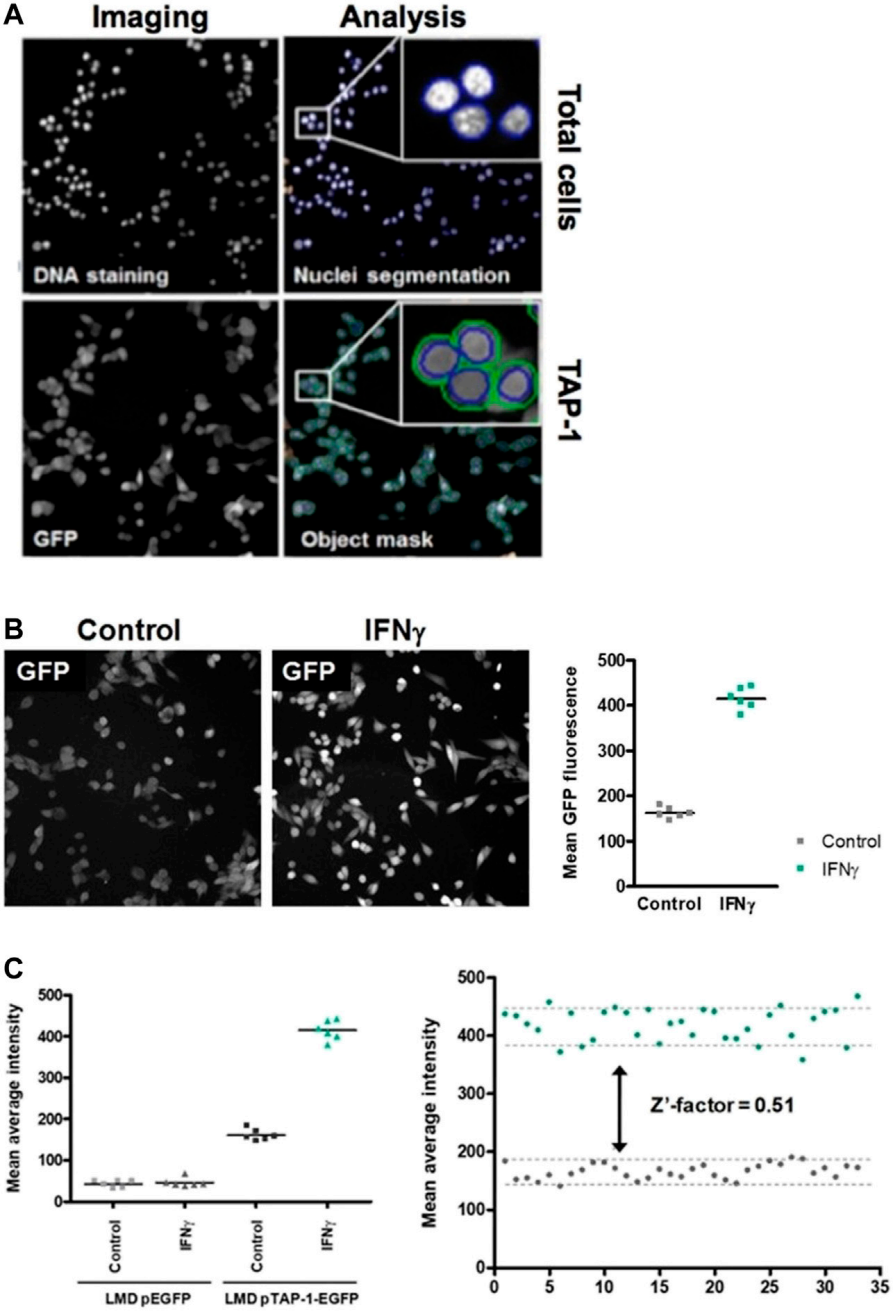
High-throughput cell-based screening assay to identify marine invertebrate extracts and pure compounds that are able to induce TAP-1 expression. **(A)** Images of the DNA staining and TAP-1 promoter-induced GFP expression in LMP pTAP-1 cells are shown. Image acquisition, segmentation and analysis of 96-well plates were carried out using the Cellomics Arrayscan VTI imager. Segmentation to delineate the nuclei based on the DNA staining fluorescence intensity was performed to identify individual objects and create a cytoplasmic mask around the nuclei in which total GFP fluorescence is measured. Average GFP fluorescence intensity and total number of cells per well were determined. **(B)** IFN-γ induces high level of GFP expression in APM-deficient LMD pTAP-1 cells. Cells were treated with 10 ng/mL of IFN-γ or 1% DMSO vehicle control. Images were taken with the same exposure time. Lines indicate the average GFP intensities. **(C)** Induction of GFP expression is TAP-1 promoter dependent. Quantitation of GFP expression in LMD pEGFP cells (LMD cells transfected with a promoterless pEGFP-1 vector) and LMD pTAP-1 EGFP cells treated with 10 ng/mL of IFN-γ or 1% DMSO vehicle control. Lines indicate the average GFP intensities. Representative graph of the Z′-factor which was calculated to assess the quality of the screening assay.

To identify candidate compounds that induce the expression of the APM component TAP-1, we developed a high-throughput cell-based screening assay using the LMD pTAP-1 (clone 15) metastatic prostate cells which allow the assessment of the induction of TAP-1 activity measured as GFP expression. The Cellomics Arrayscan VTI automated fluorescence imager was used to acquire images and determine the cell numbers based on DNA staining and the average GFP fluorescence intensity which correlates to the levels of TAP-1 induction (Figure 3A). The vehicle solution of 1% DMSO in cell culture medium was used as the negative control and IFN-γ (10 ng/mL) was used as the positive control. IFN-γ was able to induce high level of GFP expression in APM-deficient LMD pTAP-1 cells (Figure 3B) which was TAP-1 promoter-dependent (Figure 3C). To assess the quality of this assay, we determined the Z′-factor (Zhang et al., 1999) in which the dynamic range and the variation of the signal is taken into account. A Z′-factor of approximately 0.5 indicated that in this assay the dynamic range allows the clear separation between the means of positive and negative controls even when considering the variations (Figure 3C).

### Identification of marine extracts with the ability to promote upregulation of TAP-1 expression in metastatic cancer cells

**FIGURE 4:**
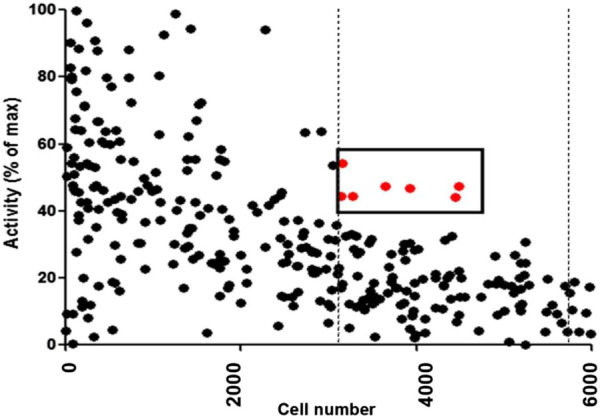
Results from high-throughput screening assay of 480 marine invertebrate extracts. The percentage of activity for each extract was calculated in comparison to the GFP fluorescence intensity of the IFN-γ positive control as described in Materials and Methods. The dots denote the results of each individual extract. Extracts with greater then 40% activity and with low cytotoxicity (cell viability within one standard deviation of 1% DMSO negative control represented by dashed vertical lines) were selected as candidates for further analysis. The red dots denote the extracts that fulfilled these criteria.

A library with a total of 480 marine invertebrate extracts estimated to contain thousands of natural products were tested at 0.175 mg/mL using the high-throughput cell-based screening assay to assess the increase of TAP-1 promoter-induced GFP expression in the LMD pTAP-1 cells. The percentage of activity for each extract was standardized to the average GFP fluorescence intensity of the 1% DMSO vehicle control and calculated in comparison to the GFP fluorescence intensity of the IFN-γ positive control as described in the Materials and Methods section. From this screening, seven marine extracts were selected based on significant TAP-1 induction (>40% activity when compared to the IFN-γ positive control) and low cell cytotoxicity (within one standard deviation of average cell density of 1% DMSO vehicle control) (Figure 4; Table 1).

**TABLE 1:** Table summarizing the plate identification (ID), well position, cell number and percentage of activity of the seven extracts that were selected for further analysis after the initial screening.

Plate ID	Well	Cell number	% Activity	Extract name
RJA UBC	B4	3,920	47	Extract 1
75966–76046				
RJA UBC	F2	3,644	47	Extract 2
75966–76046				
RJA UBC	D11	3,147	51	Extract 3
76212–76294				
RJA UBC	F8	4,447	42	Extract 4
76212–76294				
RJA UBC	D9	4,477	44	Extract 5
76295–44708				
RJA UBC	F6	3,272	44	Extract 6
76295–44708				
RJA UBC	C11	3,139	44	Extract 7
44709–55480				

### Validation of the selected marine extracts

**FIGURE 5:**
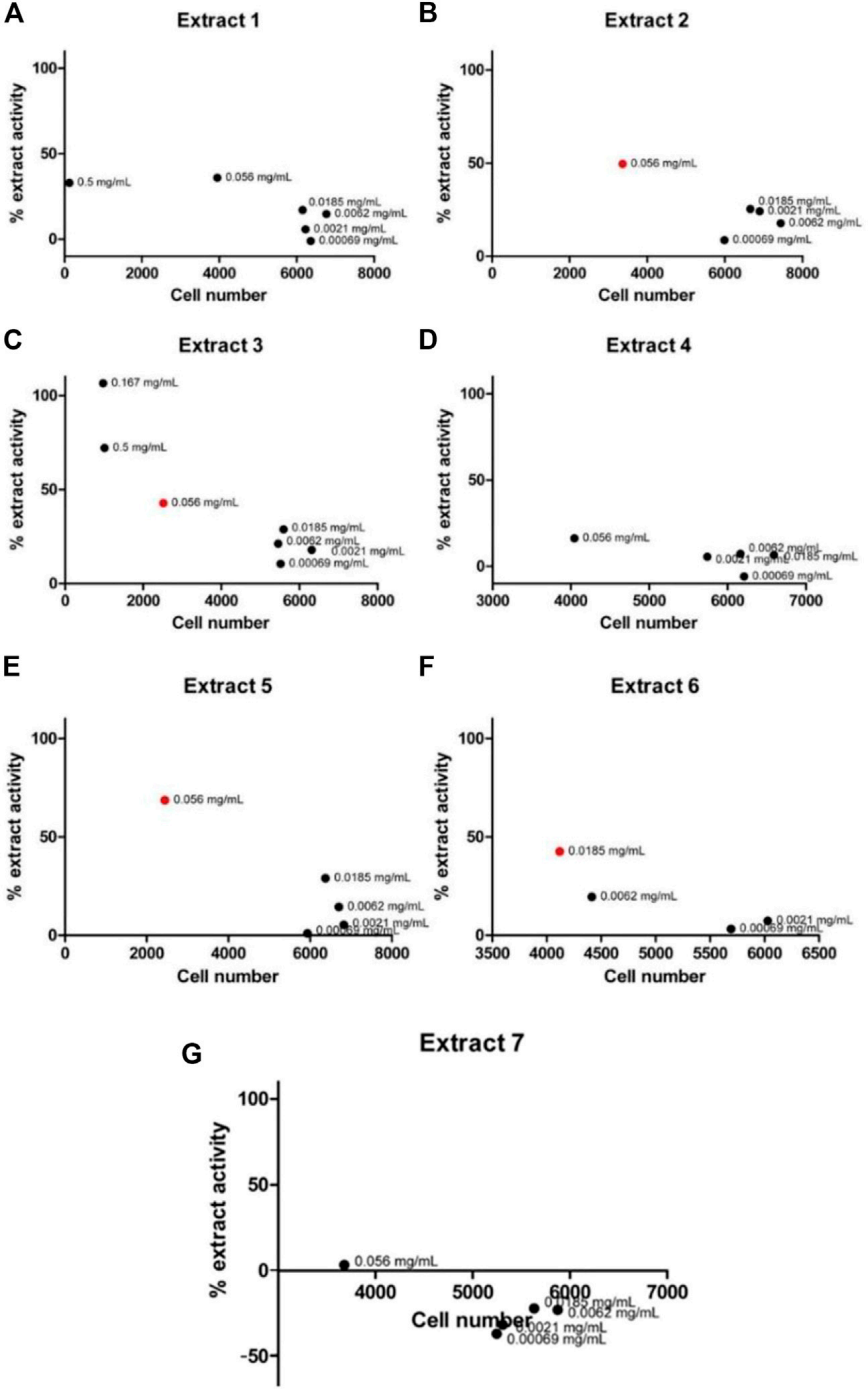
Graph of percentage of extract activity versus cell number for Extract 1 **(A)**, Extract 2 **(B)**, Extract 3 **(C)**, Extract 4 **(D)**, Extract 5 **(E)**, Extract 6 **(F)** and Extract 7 **(G)**. Selected active marine extracts were serially diluted and further tested in LMD pTAP-1 cells using the Cellomics Arrayscan. Percentage of extract activity was plotted against the average cell number to evaluate the extract dilutions that provide the highest extract activity with lowest cytotoxicity. GFP intensity measurements were not provided in wells treated with extract concentrations that showed high cytotoxicity (low cell numbers) and therefore are not shown in the graphs. Four extracts showed dilutions which were detected as active (depicted as red dots).

**FIGURE 6:**
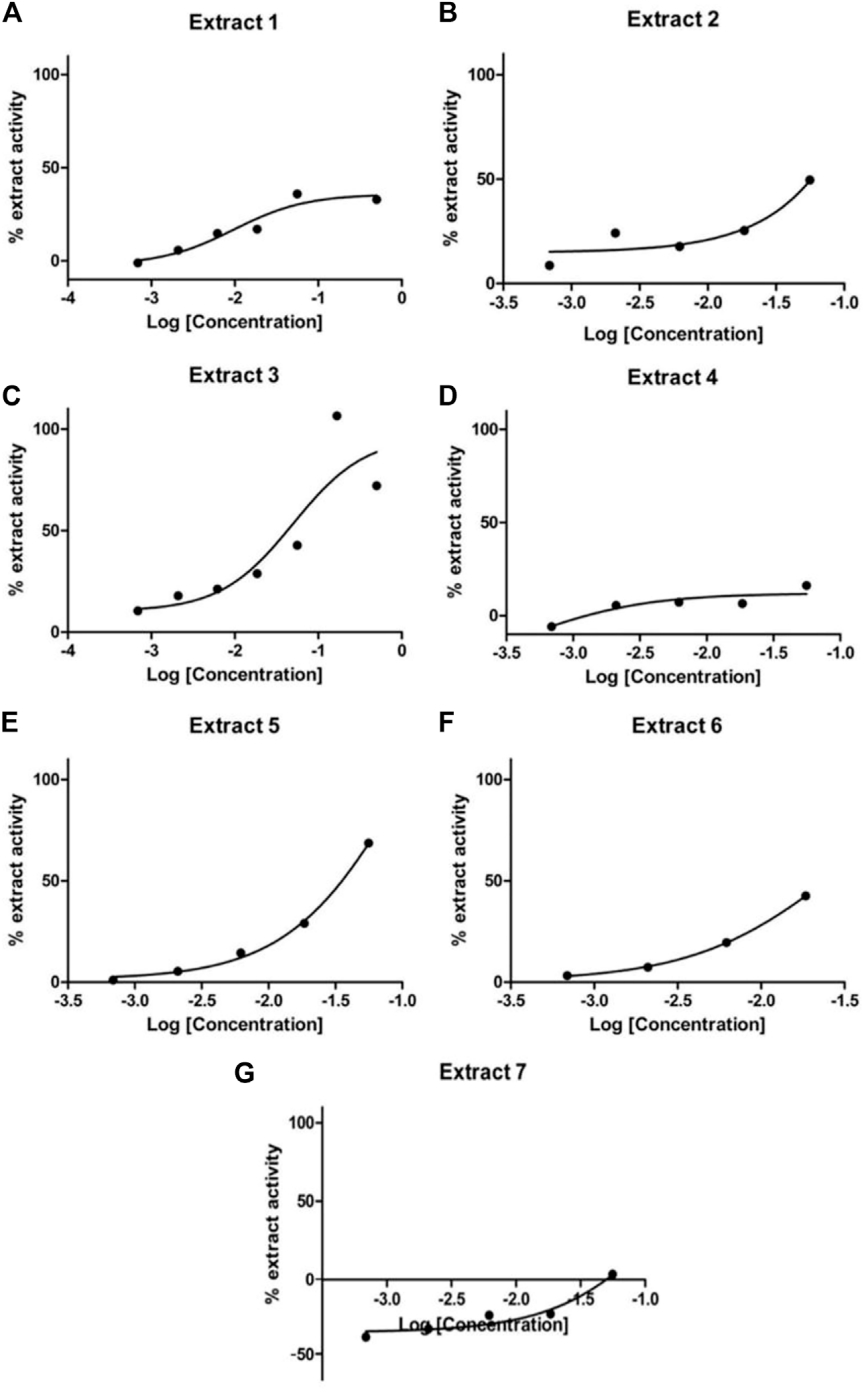
Dose-response curves for Extract 1 **(A)**, Extract 2 **(B)**, Extract 3 **(C)**, Extract 4 **(D)**, Extract 5 **(E)**, Extract 6 **(F)** and Extract 7 **(G)**. Selected active marine extracts were serially diluted and further tested in LMD pTAP-1 cells using the Cellomics Arrayscan.

**FIGURE 7:**
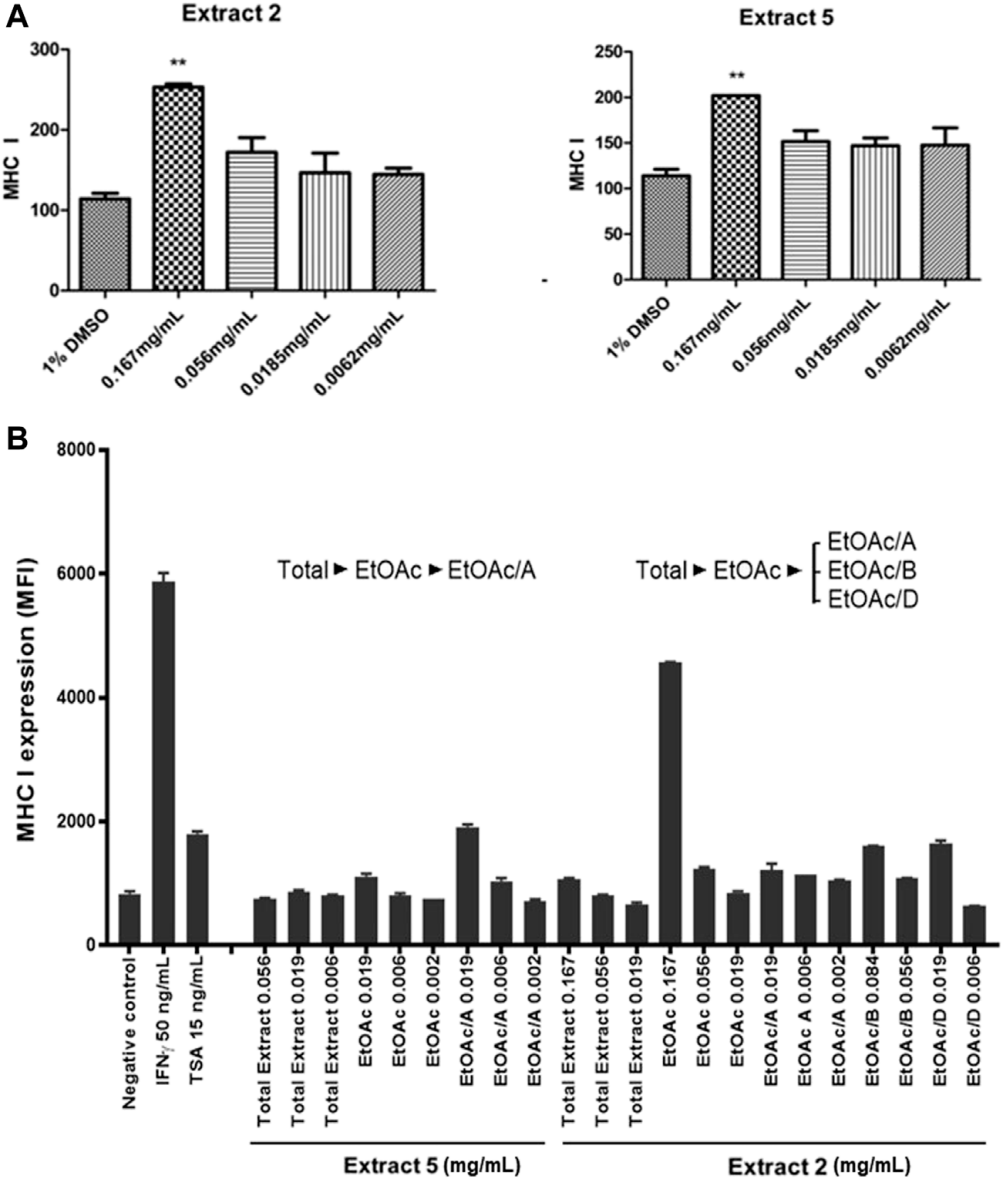
Identification of two selected marine extracts (Extracts 2 and 5) with the ability to induce MHC-I surface expression in metastatic cancer cells. **(A)** MHC-I surface expression in LMD pTAP1 metastatic cancer cells was quantified using flow cytometry upon stimulation for 48 h with Extracts 2 and 5 at varying concentrations. **(B)** Extracts 2 and 5 were fractionated to identify the components inducing the expression of MHC-I. The fractionated extracts were tested for their ability to induce MHC-I surface expression in LMD pTAP1 cells 48 h after treatment using flow cytometry. Mean fluorescence intensity (MFI); Trichostatin A (TSA).

To validate the activity of the selected marine extracts and detect the extract concentrations with low cytotoxicity and concurrently with the highest possible percentage of extract activity, the seven active marine extracts (listed in Table 1), were serially diluted (in the range of to 0.5 mg/mL to 0.00069 mg/mL) and further tested in LMD pTAP-1 cells using the Cellomics scanner. To identify these extract concentrations, diagrams with the percentage of extract activity plotted against the average cell number (Figures 5A–G) and dose-response curves in which the data were fitted into a sigmoidal curve (Figures 6A–G) were generated. Extract dilutions that lead to cell numbers below the average cell density of the negative control (cells treated with 1% DMSO) minus one standard deviation from the average were considered cytotoxic. Extract dilutions that provided results within the range of the defined cell number and with a percentage of extract activity higher than 40% are highlighted in the diagrams as a red dot. Dilutions of the seven previously detected active marine extracts resulted in four extract dilutions showing results within the described criteria to be active. The extracts that fitted these criteria were Extract 2 with 49.5% activity at 0.056 mg/mL (Figures 5B, 6B), Extract 3 with 42.8% at 0.056 mg/mL (Figures 5C, 6C), Extract 5 with 68.6% activity at 0.056 mg/mL (Figures 5E, 6E) and Extract 6 with 42.5% at 0.0185 mg/mL (Figures 5F, 6F). Extracts 1 (Figures 5A, 6A), 4 (Figures 5D, 6D) and 7 (Figures 5G, 6G) showed only marginal percentage of activity and therefore were not considered for further studies.

### Extracts 2 and 5 induce MHC-I expression at the cell surface of metastatic cancer cells

Of the four marine extracts confirmed to be active, Extract 6 was determined to be from a non-identifiable marine invertebrate and Extract 3 did not show reliable results upon retesting. Therefore, here we focused on Extract 2 and Extract 5, which showed low cytotoxicity and highest activity at the lowest concentrations and were titratable and highly replicable (Figures 5, 6). Extracts 2 and 5 were further tested for their ability to induce MHC-I expression at the cell surface in LMD pTAP-1 metastatic cancer cells using flow cytometry. Both extracts showed a significant increase in cell surface MHC-I expression (Figure 7A), making them strong candidate sources of natural product lead compounds for development of therapeutic agents to treat immuno-evasive cancers.

### Isolation of the active component from a marine extract and analysis of its chemical structure

**FIGURE 8:**
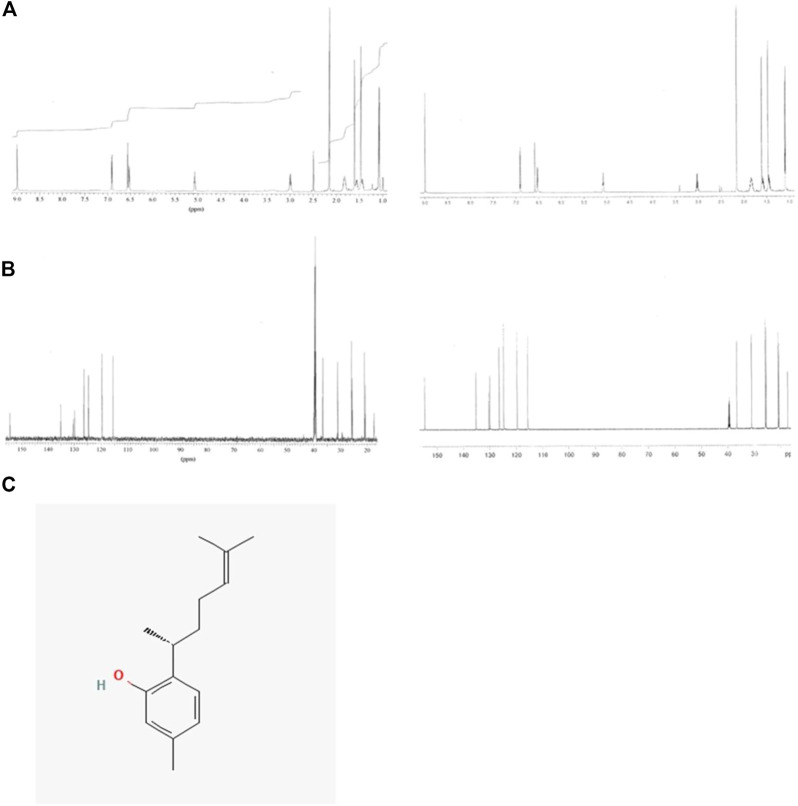
NMR spectra of the natural product curcuphenol identified in Extract 2 and synthetic curcuphenol. **(A)** Comparison of the 1H NMR spectra of the natural product (left) and synthetic curcuphenol (right) in DMSO-d6 at 600 MHz. **(B)** Comparison of the 13C NMR spectra of the natural product and synthetic curcuphenol in DMSO-d6 at 150 MHz. **(C)** Molecular structure of natural curcuphenol.

To identify the active biological components of the extracts, Extracts 2 and 5 were subjected to bioassay-guided fractionation using solvent/solvent (water/ethyl acetate) partitioning, Sephadex LH20 size separation chromatography, and high-performance liquid chromatography (HPLC). The fractions from Extracts 2 and 5 were tested by flow cytometry alongside the whole extracts for their ability to induce MHC-I surface expression. Both extracts were able to induce MHC-I expression, however, for the Extract 5 (76,336) fractions, we were unable to isolate the active compound and establish a structure. For Extract 2 (76,018, marine sponge *Halichondria* sp.), one fraction, comprising of the ethyl acetate soluble materials induced a significant increase in MHC-I expression compared to all other fractions tested (Figure 7B). Further bioassay-guided fractionation of the Extract 2 ethyl acetate soluble material gave a pure active natural product that was unambiguously identified by nuclear magnetic resonance (NMR) and mass spectrometry (MS) analysis to be curcuphenol (Figure 8).

### Curcuphenol induces MHC-I and TAP-1 expression in APM-deficient A9 metastatic lung carcinoma cells

**FIGURE 9:**
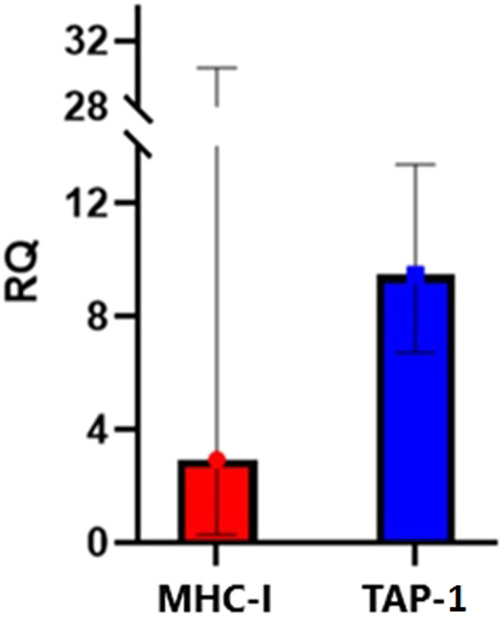
Curcuphenol induces MHC-I and TAP-1 mRNA expression in APM-deficient A9 metastatic lung cancer cells. A9 cells were stimulated with 75uM (16.37 µg/mL) of curcuphenol for 48 h. Relative quantification (RQ), including upper and lower limits, were calculated by the 7,500 Fast RT-PCR system software. Values were normalized to SDHA106 housekeeping gene and calculated with 1% DMSO treatment as reference where cells treated with 1% DMSO were normalised to 1.

Further, to confirm that curcuphenol induces endogenous APM expression, A9 metastatic lung carcinoma cells were treated with 75uM (16.37 µg/mL) of curcuphenol, and MHC-I and TAP-1 mRNA expression were quantified. Indeed, when compared to the 1% DMSO vehicle control, stimulation with curcuphenol increased expression of MHC-I and TAP-1, with an RQ of 2.93 and 9.48, respectively (Figure 9). This also provides evidence that curcuphenol induces the expression of APM components and reverse immune-escape mechanisms in metastatic tumour cells from multiple tissue origins.

## Discussion

The selective pressure of immune surveillance on genetically unstable tumour populations yields tumours that have lost expression of APM components resulting in reduced assembly of functional MHC or HLA molecules (Alimonti et al, 2000; Shankaran et al., 2001). This phenotype is often associated with immune-escape of metastatic cancers (Newman and Cragg, 2016a; Alimonti et al, 2000). Several types of cancer, including breast cancer (Alpan et al., 1996; McDermott et al., 2002), renal carcinoma (Kitamura et al., 1997), melanoma (Kamarashev et al., 2001; Chang et al., 2006), colorectal carcinoma (Cabrera et al., 2005), head and neck squamous cell cancer (Andratschke et al., 2003), cervical cancer (Mehta et al., 2008) and finally prostate carcinoma exhibit APM deficits and show a clear correlation between HLA downregulation and poorer prognosis (Blades et al., 1995; Zhang et al., 2003). Depending on the tumour type, the loss of functional HLA molecules or other APM components occurs in up to 90% of patients with metastatic disease (Blades et al., 1995; Zhang et al., 2003; Stenton et al., 2013). Subsequently, this provides tumours the ability to become unrecognizable or operationally ‘invisible’ to cytotoxic T lymphocytes, rendering them profoundly refractory to emerging immunotherapeutics such as chimeric antigen receptor-T cells and immune checkpoint blockage inhibitors. Currently, only 15%–30% of patients respond to existing immunotherapies (Seliger, 2016). Discovering new therapeutic candidates that overcome immune-escape and augment the emerging immunotherapy modalities should be a priority.

Recently, cancer cells subjected to selection for increased or decreased expression of MHC-I have been subjugated to forward genetic screenings, identifying a substantial number of new genes that are able to regulate MHC-I antigen presentations, including IRF2, PBAF, PRC2, and SPPL3 (Kriegsman et al., 2019; Patel et al., 2017; Burr et al., 2019; Chang et al., 2005; Manguso et al., 2017; del Campo et al., 2009; Del Campo et al., 2014; Garcia-Lora et al., 2003; del Campo et al., 2014). Furthermore, a CRISPR-cas9 screening in B cell lymphoma cell lines identified approximately 200 genes that may influence MHC-I expression (Almeida et al., 2021). Approximately 30 of these genes relate to CD8 T lymphocyte infiltration in multiple cancers including 10 negative-regulatory genes which were correlated with less tumor infiltrating CD8 T lymphocytes and 20 positive-regulatory genes which were correlated with more infiltrating CD8 T lymphocytes. The field can anticipate many more studies on the function of these genes in MHC-I antigen presentation and cancer immune evasion however, there have been a dearth of screens to identify chemical compounds that modify MHC-I expression.

Natural product libraries offer an excellent source of new compounds that have potential as lead compounds for development of novel chemotherapeutic agents or as cell biology tools. Extracts may be isolated from common components of food such as spices and herbs or they may come from more distant resources, such as the depths of the oceans. While spices are typically thought of as staples in cooking, there have been numerous spices identified that possess medicinal properties, including cumin, saffron, turmeric, green and black tea and flaxseed that contain curcumin (Thompson et al., 1996; Busquets et al., 2001; Abdullaev, 2002; Lai and Roy, 2004; Mason and Thompson, 2014; Sharifi-Rad et al., 2020). Another common source of natural therapeutics is herbs, which are a rich source of secondary metabolites including: polyphenols, flavonoids and brassinosteriods (Greenwell and Rahman, 2015). However, of all the natural resources, the marine environment dominates in diversity of both biologics and chemicals (Simmons et al., 2005; Gomes et al., 2016). Therefore, screening of extracts from natural marine resources is an important source of chemical inspiration for the development of novel therapeutics that may reduce cancer growth and metastasis.

Currently, this is the only description of a high-throughput cell-based screening assay designed to identify chemical compounds that induce the expression of the APM components. The new assay was used to screen a marine invertebrate natural product extract library resulting in several promising hits. In this regard, we validated the screening by demonstrating the increase of TAP-1 and MHC-I expression in metastatic carcinoma cells in response to the chemical entities we discovered. Bioassay-guided fractionation of the sponge *Halichindria* sp. lead to the isolation and identification of curcuphenol as the active compound in this extract. Curcuphenol significantly increased the expression TAP-1 and MHC-I in metastatic carcinoma cells from both the prostate and lung while at the same time exhibiting low general cytotoxicity. However, while curcuphenol can be found in nature as one of two enantiomers: S- (+) and R- (−) curcuphenol, we have not identified the enantiomeric purity or the absolute configuration of the curcuphenol isolated from the sponge extract in this study. Future work will address which of the enantiomers acts to reverse the cancer immune-escape phenotype associated with the metastatic transition of tumours.

Finally, to contextualize the significance of these discoveries, many previous studies have described an anticancer phenomenon involving the application of traditional medicines. Perhaps the leading example of this are spices such as turmeric used in the preparation of food that thought to contain an active yet uncharacterised anticancer component (Nagahama et al., 2016; Nair et al., 2019; Ojo et al., 2022). Here we document that one such active molecule is curcuphenol. Thus, this study highlights the potential medicinal value of common components of spices used in the preparation of foods or as dietary supplements and advanced nutraceuticals, for harnessing the power of the immune system to recognize and destroy metastatic cancers.

## Materials and methods

### Marine extract collection

The marine extract collection was prepared from over 5,000 frozen mollusk, tunicate, and sponge specimen samples collected by SCUBA diving at 0–40 m depths at locations tagged with a global positioning system (GPS) in regions of high marine biodiversity in Canada (Pacific coast), South Africa, the Philippines, Norway, Papua New Guinea, Indonesia, Thailand, Sri Lanka, Dominica, and Brazil. Specimens were frozen immediately after collection in the field and transported frozen to the laboratory in Vancouver. One hundred grams of each frozen specimen sample was thawed and either directly extracted with methanol or ethyl acetate or first lyophilized followed by extraction with methanol or ethyl acetate. Two milligrams of each concentrated crude methanol extract was then dissolved in 100% DMSO and stored in covered 96-well plates at −20°C. From this collection, 480 crude marine invertebrate extracts were used for the *in vitro* screening assays.

### Cell lines

#### TC-1 and A9 murine lung tumour cell lines

The TC-1 cell line originated from primary mouse lung epithelial cells (C57BL/6 background) which were immortalized using the amphotropic retrovirus vector LXSN16 carrying Human Papillomavirus E6/E7, and then transformed with pVEJB plasmid expressing the activated human c-Ha-ras oncogene. TC-1 cells form slow-growing tumours in animals and display high expression of TAP-1 and MHC-I. The cell line A9 was derived from the TC-1 tumour cell line by immunoselection *in vivo* (Smahel et al., 2003). A9 cells display low surface MHC-I (H2-K1) expression and have been shown to be metastatic in a mouse model (Saranchova et al., 2016). The cells were cultured as previously described (Smahel et al., 2003).

#### PA and LMD murine prostate carcinoma cell lines

PA and LMD cell lines are an antecedent model of non-metastatic and metastatic murine prostate cancer, respectively. PA is a murine prostate cancer cell line derived from a 129/Sv mouse, that displays a high surface expression of MHC-I. LMD is a metastatic cell line derived from PA and is deficient in the expression of TAP-1 and MHC-I (Lee et al., 2000). These cell lines were originally provided by Dr. T.C. Thompson, Baylor College of Medicine, Houston. They were cultured as previously described (Lee et al., 2000).

#### LMD reporter cell line

LMD pTAP-1 reporter cells are a TAP-1-deficient prostate carcinoma cell line that express enhanced green fluorescent protein (EGFP) under the TAP-1 promoter (Setiadi et al., 2005). LMD pTAP-1 cells were maintained in DMEM supplemented with 10% fetal bovine serum. For selection of transfected cells, 1 mg/mL geneticin was added to the tissue culture media. The cells show low GFP intensity in non-stimulated conditions and high GFP intensity upon IFN-γ stimulation. To obtain LMD pTAP-1 single cell clones, unsorted LMD pTAP-1 cells were activated with 100 ng/mL IFN-γ, and single cells with the highest GFP expression were sorted in 96-well plates. GFP fluorescence intensity in each clone was analyzed by Cellomics Arrayscan VTI automated fluorescence imager and LMD pTAP-1 clones number 14, 15 and 20 which showed low basal GFP expression were selected. These clones were transferred into larger plates until enough cells were obtained for analysis and then stimulated with different concentrations of IFN-γ. Based on the strong GFP expression upon stimulation, clone 15 was selected for further experiments.

### Cell-based screening assay

LMD pTAP-1 (clone 15) cells were seeded in 96-well plates (PerkinElmer View) at the optimized cell density of 3.5 × 10^3^ cells per well. Twenty-four hours after seeding, cells were cultured in the presence of the indicated concentrations of the marine extracts, 10 ng/mL of IFN-γ (R&D Systems) or 1% DMSO control. Plates were incubated for 48 h at 37°C in a 5% CO_2_ incubator. The medium was removed and cells fixed with 4% (v/v) paraformaldehyde containing 500 ng/mL Hoechst 33,342 (Molecular Probes). Fixed cells were stored in PBS at 4°C until further analysis. Image acquisition, segmentation and analysis of microplates were carried out using the Cellomics Arrayscan VTI automated fluorescence imager (Thermo Fisher Scientific). Images from 12 fields were acquired using a ×20 objective in the Hoechst and GFP (XF-100 filter) channels (auto-focus, fixed exposure time). The target activation algorithm was used to identify the nuclei based on Hoechst fluorescence intensity, apply a cytoplasmic mask and quantitate GFP fluorescence intensity within the cytoplasmic mask area. Average GFP fluorescence intensity (intensity per cell per pixel) and total number of cells per well were determined. To assess the quality of the screening assay, the Z′-factor (Zhang et al., 1999) was calculated as 1-[3*(δp+δn)/(|µp-µn|)], where µp, δp, µn and δn are the means (µ) and standard deviations (δ) of both the positive (p) and negative (n) controls (10 ng/mL IFN-γ and 1% DMSO, respectively). The percentage of activity for each extract was calculated according to the equation 100*[(x-µn)/(µp-µn)] where x is the average GFP fluorescence intensity of each extract tested and, µp and µn are the average GFP fluorescence intensities of the 10 ng/mL IFN-γ positive control and the 1% DMSO negative control, respectively.

### Screening of marine extract library

To identify marine extracts with potential to upregulate TAP-1 expression in the LMD pTAP-1 cells, the marine extracts were tested at 0.175 mg/mL using the cell-based screening assay. In pre-screenings, the extracts were thawed, rocked for 2 h, and transferred to tissue culture plates with a pin robot. Based on the percentage of activity and cell viability for each extract, the pre-screenings identified seven potentially active extracts. These potentially active extracts were further examined with Cellomics Arrayscan. Extracts dilutions were performed to identify the extract concentration with the highest percent activity. The extracts were stored at −20°C and thawed at room temperature. After thawing, the extract plates were rocked for 2 h. The extracts were serially diluted to 0.5 mg/mL, 0.167 mg/mL, 0.056 mg/mL, 0.0185 mg/mL, 0.0062 mg/mL, 0.0021 mg/mL and 0.00069 mg/mL, and added 24 h after plating the cells in 96-well plates. Following a 48 h incubation at 37°C in 5% CO_2_, the cells were fixed as described earlier, Hoechst stained and analyzed with Cellomics Arrayscan.

### Evaluation of MHC-I surface expression by flow cytometry

LMD pTAP-1 cells were plated in 6-well plates at a concentration of 1 × 10^3^ cells per well in a 2 mL volume. The following day, cells were treated with the indicated concentrations of the compounds and incubated for 48 h at 37°C. After incubation, the cells were trypsinized, washed and stained with APC-conjugated anti-mouse MHC-I (anti-H-2Kb, Biolegend) and assessed by flow cytometry analysis. As a positive control, cells were treated with either 50 ng/mL of IFN-γ or 15 ng/mL of trichostatin A (TSA, Sigma-Aldrich) for 48 h, to induce surface MHC-I expression, and vehicle alone (1% DMSO) was used as a negative control.

### Isolation of active component from marine extract and analysis of its chemical structure

#### Marine extract fractionation

To obtain pure active natural products for further biological examination, extracts showing potential activity were subjected to bioassay-guided fractionation using solvent/solvent (water/ethyl acetate) partitioning, Sephadex LH20 size separation chromatography, and high-performance liquid chromatography (HPLC). Fractionation was performed in multiple rounds to ultimately identify a single active compound. Each sub-fraction was then tested using the cell-based screening assay to identify the active sub-fraction and then further analyzed by flow cytometry to verify MHC-I surface expression.

#### Isolation of curcuphenol from extract 2 (76018)

Specimens of the massive orange sponge, *Halichondria* sp., were collected by hand using SCUBA at Solong-on, Siquijor Island, Philippines (09o 10′N, 123o 29′ E). A voucher sample has been deposited at the Netherlands Centre for Biodiversity Naturalis in Leiden, the Netherlands (voucher number: RMNH POR. 5872). Lyophilized sponge material (15 g) was extracted with methanol (3 × 50 mL) at room temperature. Bioassay-guided fractionation of the crude methanol extract identified curcuphenol as the active component. Analysis of the 1D and 2D nuclear magnetic resonance (NMR) and mass spectrometry (MS) data collected for the curcuphenol sample obtained from the *Halichondria* sp. unambiguously identified its constitution, but its absolute configuration was not determined.

### TAP-1 and MHC-I mRNA expression in A9 cells

#### Cell treatment

A9 cells were seeded at 3 × 10^5^ onto a 6-well plate in 2 mL of DMEM. Twenty-four hours after seeding, cells were stimulated for 48 h with one of the following treatments: 1% DMSO in DMEM as a vehicle control, 75uM (16.37 µg/mL) of curcuphenol, or 20 ng/mL of IFN-γ as a positive control. Curcuphenol stock was dissolved in DMSO. All compounds were diluted in 1% DMSO.

#### Reverse transcription and real-time PCR

RNA was isolated using the PurelinkTM RNA Mini Kit (Invitrogen), followed by DNase I (Ambion) treatment. The primers used for this experiment can be found in the Supplementary Material (Appendix 1). RNA was then reverse transcribed into cDNA using the SuperScript^®^ III First-Strand Synthesis System for RT-PCR (Invitrogen). Real-time PCR (RT-PCR) was done using a 7,500 Fast RT-PCR system (Applied Biosystems) with the following parameters: 40 cycles (95°C denaturing 15 s, 60°C annealing for 1 min). Relative quantification (RQ), including upper and lower limits, were calculated by the 7,500 Fast RT-PCR system software. Values were normalized to the housekeeping gene SDHA106 and calculated with 1% DMSO treatment as reference where cells treated with 1% DMSO were normalised to 1.

### Statistical analysis

Data were analyzed with R and Excel. A Student’s *t*-test was used for determining statistical significance between controlled test groups; *p* ≤ 0.05 was considered significant.

## Data Availability

The raw data supporting the conclusion of this article will be made available by the authors, without undue reservation.

## Appendix 1 – Primer sequences for RT-PCR

**Table:**

**Gene**	**Oligo sequences**	
SDHA	Forward	5’-CTT GAG ATC CGT GAA GGA AGA G-3’
	Reverse	5’-CAC CTG TCC CTT GTA GTT AGT G-3’
MHC I (H2Kb)	Forward	5’-TCG CTG AGG TAT TTC GTC-3’
	Reverse	5’-TTG CCC TTG GCT TTC TGT-3’
TAP1	Forward	5’-TTA TCA CCC AGC AGC TCA GCC T-3’
	Reverse	5’-TCA GTC TGC AGG AGC CGC AA-3’
LMP	Forward	5’-CGA CAG CCC TTT ACC ATC G-3’
	Reverse	5’-TCA CTC ATC GTA GAA TTT TGG CAG-3’
