# Erratum: A novel cell-based screen identifies chemical entities that reverse the immune-escape phenotype of metastatic tumours

**DOI:** 10.3389/fphar.2023.1233717

**Published:** 2023-06-12

**Authors:** Frontiers Production Office

**Affiliations:** Frontiers Media SA, Lausanne, Switzerland

**Keywords:** antigen processing machinery, curcuphenol, major histocompatibility complex class I, metastatic tumours, natural products, high throughput cell-based assay, drug discovery

Due to a production error, “Eliana Al Haddad” and “Kyong Bok Choi” were not included as authors in the published article. The corrected **Author contributions** and **Acknowledgments** statements appear below.

